# P03 Uncovering the driver of a fatal disease

**DOI:** 10.1093/rap/rkae117.034

**Published:** 2024-11-05

**Authors:** Jasiththa Anpalagan, KyawNaing Oo, Sarah Bingham

**Affiliations:** Queen Elizabeth Hospital, King’s Lynn, United Kingdom; Queen Elizabeth Hospital, King’s Lynn, United Kingdom; Queen Elizabeth Hospital, King’s Lynn, United Kingdom

## Abstract

**Introduction:**

Haemophagocytic lymphohistiocytosis (HLH) is a rare, critical condition with 30-40% mortality, marked by excessive immune activation, severe systemic hyperinflammation, elevated cytokines, and multiorgan failure. HLH is classified into primary and secondary types. Primary HLH, typically in children, results from genetic mutations impairing natural killer and T cells. Secondary causes include rheumatological conditions, malignancies, infections, and treatments for malignancies. Common rheumatological causes are Still’s disease, lupus, rheumatoid arthritis and vasculitis. Sarcoidosis is an exceptionally rare cause, documented only in case reports. Diagnosis was delayed in this case due to granulomatous lesions masking an underlying lymphoma.

**Case description:**

A 65-year-old woman presented to the haematology clinic with a three-month history of unexplained fatigue and cytopenia, despite normal hematinic levels. Imaging revealed splenomegaly and generalised small-volume lymphadenopathy, prompting investigation for lymphoproliferative disease. However, bone marrow trephine and biopsy were inconclusive for myelodysplasia or lymphoproliferative disorders. Positive ANA and Ro antibodies led to a referral to rheumatology, though further assessment showed no evidence of autoimmune disease.

Multiple hospital admissions were required due to neutropenic sepsis and ongoing cytopenia needing blood transfusions, despite negative infection screenings. A splenic biopsy confirmed non-caseating granulomatous inflammation with elevated calcium, angiotensin-converting enzyme, and vitamin D levels supporting a diagnosis of sarcoidosis, despite minimal clinical symptoms. After excluding infectious causes of granulomas, she was treated with prednisolone for sarcoidosis-related cytopenia. However, cytopenia recurred with prednisolone tapering, necessitating readmission with severe cytopenia, high fever, and ferritin levels exceeding 20,000, fulfilling HLH criteria prompting immediate treatment with anakinra.

Her case was urgently discussed at the national HLH and regional rare autoimmune disease meetings. Treatment with anakinra, steroids, and IV immunoglobulin stabilised her condition, and she was discharged with a plan to taper anakinra and steroids. A repeat bone marrow biopsy during this admission revealed multiple non-caseating granulomas with increased erythrophagocytic activity and a 30% population of highly proliferative EBV-negative polymorphous B cells. Further analysis confirmed EBV-negative high-grade B-cell lymphoma six weeks after the diagnosis of HLH. Her condition became critical again before the confirmation of lymphoma on weaning dose of anakinra, requiring readmission with a relapse of HLH. The relapse was managed with anakinra accordingly.

The haematology and regional lymphoma multidisciplinary team (MDT) initiated chemotherapy for her lymphoma. She responded well to the first cycle, with significant improvement in cell counts and ferritin levels. Later, the anakinra and steroids were tapered completely. She is currently in good health.

**Discussion:**

Our patient’s journey was fraught with diagnostic challenges from the outset. Initial presentations of cytopenia, generalised lymphadenopathy, and massive splenomegaly strongly suggested lymphoma. However, histological confirmation of lymphoma was absent in both splenic and bone marrow biopsies. The splenic biopsy revealed a non-caseating granuloma which, combined with elevated ACE and high calcium and vitamin D levels, led us to diagnose sarcoidosis although isolated haematological involvement in sarcoidosis is highly unusual.

Given the worsening cytopenia, treatment for sarcoidosis was commenced. Initially, the patient’s cell counts improved, but relapsing cytopenia occurred while tapering the treatment regimen. The poor response, even with high steroid doses, prompted a broader re-evaluation of the diagnosis. Previous instances of elevated ferritin levels were dismissed as an inflammatory response, but retrospectively, it became clear that the patient had HLH well before it was accurately diagnosed and was only partially treated with steroids.

This case underscores the crucial importance of considering high ferritin levels, regardless of the clinical context. Additionally, an inadequate response to treatment should prompt a reconsideration of the diagnosis. A repeat bone marrow biopsy, conducted nearly four months after the initial one, confirmed the diagnosis of high-grade B cell lymphoma.

Upon retrospective analysis, there are case reports of granulomatous lesions obscuring an underlying lymphoma. From the beginning, we encountered numerous misleading indicators such as elevated ACE levels, granulomatous lesions and high vitamin D levels. Isolated haematological involvement, massive splenomegaly, and generalized lymphadenopathy without typical sarcoid involvement were all atypical for sarcoidosis. When confronted with numerous atypical symptoms, a diligent search for alternative diagnoses is imperative.

This case highlights the intricacies and challenges in diagnosing rare diseases like HLH and emphasises the need for vigilance and thorough investigation when typical presentations are absent.

**Key learning points:**

• First, recognising and accepting diagnostic uncertainties while ensuring patients’ safety is important. The diagnostic process has been challenged and complicated throughout this patient’s journey with numerous uncertainties and multiple red herrings. High ACE, elevated 1,25OH vitamin D, and calcium levels are nonspecific but must be considered significant in the presence of non-caseating granulomatous lesions. The initial bone marrow biopsy ruled out haematological or lymphoproliferative malignancy. Despite the rarity of pure haematological involvement in sarcoidosis, the working diagnosis of sarcoidosis was made.

• Secondly, it is essential to understand prompt treatment and immediate intervention with high-dose steroids for worsening cytopenia as life-saving management. It is also crucial to involve expert MDT discussions when faced with diagnostic uncertainty. These consultations can provide valuable insights and guide further investigations.

• In addition, when conventional therapy does not yield the expected response, it is essential to revisit and extensively search for the underlying diagnosis. Although initially treated for sarcoidosis, the ongoing search for an underlying malignancy continued throughout the course, which leads to the final diagnosis

• Moreover, HLH is a rare but potentially fatal disease. Despite sarcoidosis being an extremely rare cause of HLH, active search for HLH in this case tremendously improved the patient’s survival chances. It is vital to promptly treat patients when the risks of non-treatment outweigh the benefits of delayed treatment or further evaluation. A repeat bone marrow biopsy was scheduled before administering IV methylprednisolone to ensure the treatment did not affect the diagnostic yield

• Finally, there are case reports of granulomatous lesions masking lymphoma. The granuloma load can conceal underlying tumour cells. Granulomas can lead to increased production of vitamin D and subsequently calcium. High ACE levels are also known in lymphoma. Despite nonspecific findings, the patient was started on treatment due to clinical deterioration.

